# Exploring the socio-emotional factors associated with subjective well-being in the unemployed

**DOI:** 10.7717/peerj.2506

**Published:** 2016-10-06

**Authors:** M. Pilar Berrios, Natalio Extremera, M. Pilar Nieto-Flores

**Affiliations:** 1 Department of Psychology, University of Jaén, Jaén, Spain; 2 Department of Social Psychology, University of Málaga, Málaga, Spain

**Keywords:** Unemployed, Well-being, Social support, Depression, Emotional intelligence, Stress

## Abstract

In this study, we examined the relations between dimensions of Perceived Emotional Intelligence (PEI) and classic constructs, such as social support, on depression, stress, and subjective well-being indicators (life satisfaction and happiness). The study also sought to determine whether PEI dimensions accounted for a significant portion of the variance beyond that of classic constructs in the study of depression, stress, and well-being outcomes in a sample of 442 unemployed subjects. Results indicated that social support and all PEI dimensions are found to be significant and negatively related to depression and stress, and these variables were also found to be significant and positively associated with life satisfaction and happiness. Additionally, results using regression analysis indicated that PEI, and specifically use of emotions and regulation of emotions, explain a significant amount of the variance of all outcomes after controlling for socio-demographics and social support dimensions. Finally, theoretical and practical implications of these constructs and their relation with psychological adjustment and well-being in unemployed people are discussed.

## Introduction

Unemployment remains a major economic and social problem in the European Union. According to the Eurostat database, the unemployment problem has been most acute in Greece, Spain, Portugal, and Italy (Eurostat Unemployment Database, 2016). In 2016 Spain has the second highest European Union unemployment rate, after Greece. In short, the national unemployment rate in the first quarter of 2016 for Spain was 21% with 4,791,400 individuals unemployed (INE Instituto Nacional de Estadística, 2016). Therefore, unemployment is one of the biggest problems for European countries, not only because of its socio-economic impact, but also because of its psychological consequences. The psychosocial impact of job loss is considered to be a major stressful event with long-term consequences for individuals (Wanberg, 2012). Accordingly, it is well documented that job loss is associated with a significant increase in psychological distress, depression, and worsening mental health (Paul & Moser, 2009; Stankunas et al., 2006), and it is associated with a significant decrease in psychological well-being (McKee-Ryan et al., 2005). Therefore, analysis of personal resources that help to improve the experience of unemployment remains an issue of great interest to researchers and career counsellors (Rey, Extremera & Peláez-Fernández, 2015). One of the main avenues of research has focused on the role of cognitive–affective variables, which would facilitate coping with these demands and would increase the levels of well-being (McKee-Ryan et al., 2005). Numerous psychosocial predictors of mental health and well-being in unemployment have been examined to better understand this relationship. These resources will ameliorate the potentially negative consequences of unemployment and consequently provide protection. Social support and Emotional Intelligence (EI) might be considered two of these psychosocial resources.

### Social support, psychological adjustment and well-being

Social support can be defined as the perception or experience that one is loved and cared for, esteemed, and valued, and part of a social network of communication and mutual assistance (Wills, 1991). A consistent body of research has emerged over recent decades to show that close relations with family, friends, and significant others is a protective factor that helps guard against the deleterious mental and health effects of unemployment (Bjarnason & Sigurdardottir, 2003). Thus, lack of social support is positively related to depressive and anxious symptomatology, both in the general population (Siedlecki et al., 2014) and among the unemployed population (Rey, Extremera & Peláez-Fernández, 2015). Similarly, social support has been found to have a particularly marked effect on how well individuals cope with unemployment, that is, unemployed people cope with the loss of a job more successfully if they have a wide social support network (Bjarnason & Sigurdardottir, 2003; Ślebarska, Moser & Gunnesch-Luca, 2009).

In sum, the adverse impact of unemployment on the psychological adjustment and well-being of unemployed individuals in supportive social contexts might be less than its effect on unemployed individuals living in less supportive contexts (Rey, Extremera & Peláez-Fernández, 2015).

### Emotional intelligence, psychological adjustment and well-being

There are currently two predominant models of EI: mixed and ability models (Mayer, Roberts & Barsade, 2008). Mixed models describe EI as a broad conception of intelligence that combines social skills, traits, and dispositional behavior. On the other hand, Mayer & Salovey (1997) ability model of EI involves the ability to carry out accurate reasoning about emotions and the ability to use emotions and emotional knowledge to enhance thought, thus enabling the subject to solve social problems and to adapt effectively to the environment (Mayer, Roberts & Barsade, 2008). In the operational definition of EI, these authors distinguish four specific skills: (1) the ability to identify and express emotions; (2) the ability to use emotions in decision-making; (3) the ability to understand emotions, and; (4) the ability to regulate emotions, both in oneself and in others.

A recent line of research has focused on the unique contributions of EI in explaining the psychological adjustment and well-being of different collectives controlling for classic constructs (Zeidner, Matthews & Roberts, 2012). The results of this research indicate that EI is negatively related to stress and depression (Augusto-Landa et al., 2008; Salguero, Extremera & Fernández-Berrocal, 2012), and positively related to various indicators of well-being (Gallagher & Vella-Brodrick, 2008; Por et al., 2011). All these findings were obtained in samples of the general population, but data on these relationships for unemployed people remain scarce. Prior studies have found that self-regulatory processes may exert influence on both longer-term affective states and on the mental health of individuals who lose their jobs (Wanberg et al., 2012). These results suggest that affective and personal resources are important to subjective well-being and psychological adjustment during unemployment (McKee-Ryan et al., 2005).

In sum, social support and EI might be resources that can help individuals to better cope with stressful situations, such as unemployment, and to increase psychological adjustment and well-being.

### Motivation for the present study

Previous literature has confirmed empirical evidence of the relationship between social support and psychological adjustment and subjective well-being in unemployed people. Despite these findings, researchers have not examined the joint contribution of social support and EI dimensions to well-being and psychological adjustment during unemployment. These findings might be significantly useful in the design and development of employment promotion programs and clinical interventions with unemployed people.

Given the aforementioned considerations, the purpose of this study was twofold. The first purpose was to examine the relationships between social support, EI dimensions, stress, depression, and indicators of subjective well-being (life satisfaction and happiness) in a sample of unemployed people. Second, we examined the incremental validity of EI dimensions on stress, depression, and well-being outcomes beyond what is accounted for by the influence of social support. According to the aforementioned studies, higher levels of social support and EI dimensions are expected to be negatively associated with psychological distress and depression and positively associated with well-being outcomes. Thus, our study hypothesized that EI dimensions will explain further additional variance in psychological distress, depression, and well-being outcomes after removing any variance explained by social support.

## Materials and Methods

### Participants

The sample was composed of 442 unemployed people (225 males and 217 females) from two southern cities in Spain, who participated voluntarily and anonymously in the study. The mean age was 32.2 years (SD = 9.9). The average duration of unemployment was 20.28 months (SD = 30.7 months). The marital status of the participants was: 59.8% single, 13.5% married, 1.5% divorced, 16.2% widow(er), and 9% cohabiting.

### Instruments

#### Social support

The Multidimensional Scale of Perceived Social Support (MSPSS) (Zimet et al., 1988) consists of twelve items relating to perceived social support and is answered on a seven-point Likert-type scale, ranging from 1 (strongly disagree) to 7 (strongly agree). Three separate scores can be calculated for the sources of support: (1) Significant Other; (2) Family; and (3) Friends, which can be added together to give a total social support score. Higher scores indicate greater perceived social support. We used the validated Spanish version (Landeta & Calvete, 2002).

#### Emotional intelligence

The Wong and Law Emotional Intelligence Scale (WLEIS) (Wong & Law, 2002) was used to measure EI abilities. This self-report measure is based on the definition of EI proposed by Salovey & Mayer (1990) and consists of four dimensions: (1) self-emotion appraisal; (2) other-emotion appraisal; (3) use of emotion; and (4) regulation of emotion. Each subscale consists of four items with a seven-point response format, ranging from 1 (strongly disagree) to 7 (strongly agree). The scale includes items such as: “I am quite capable of controlling my own emotions.” This version of WLEIS has been shown to have good validity and reliability in Spanish populations (Pena, Rey & Extremera, 2012; Rey & Extremera, 2011).

#### Depression, anxiety and stress

The Depression Anxiety Stress Scale-21 (DASS-21) (Lovibond & Lovibond, 1995), which aims to measure psychological distress was developed according to the tripartite model of anxiety and depression and is a set of three self-report scales designed to measure the negative emotional states of depression, anxiety, and stress. Each of the three DASS scales contains seven Likert-type scales. Alpha coefficients are all above 0.85 and validity has been supported through its correlation with other measures of depression and anxiety (Lovibond & Lovibond, 1995). The Spanish version showed satisfactory internal consistency, convergent validity, and an acceptable divergent validity (Bados, Solanas & Andrés, 2005). In our study, we used stress and depression subscales.

#### Life satisfaction

We used the Spanish version (Atienza, Balaguer & García-Merita, 2003) of the Satisfaction With Life Scale (SWLS) (Diener et al., 1985) to assess perceived global life satisfaction. This scale comprises five self-referencing statements and requires subjects to rate the extent to which they agree or disagree with each statement on a seven-point scale (1 = strongly disagree to 7 = strongly agree). Both English and Spanish versions have shown evidence for discriminant validity and appropriate internal consistency (Atienza, Balaguer & García-Merita, 2003; Diener et al., 1985).

#### Happiness

The Subjective Happiness Scale (SHS) (Lyubomirsky & Lepper, 1999) is a widely used, 4-item global assessment of happiness. Two items request respondents to describe themselves using both absolute ratings and ratings relative to peers, whilst the other two items offer brief descriptions of happy and unhappy individuals and ask respondents about the extent to which each description describes them. Each item was assessed on a seven-point Likert scale (e.g., “In general I consider myself:” 1 = Not a very happy person to 7 = A very happy person). Across 14 samples, the SHS has demonstrated good psychometric properties, such as test–retest reliability, discriminant validity, and convergent validity (Lyubomirsky & Lepper, 1999). Furthermore, the Spanish SHS has recently been translated into Spanish with satisfactory psychometric qualities. We used a well-validated Spanish version (Extremera & Fernández-Berrocal, 2014).

### Procedure

Participants who were utilizing the national employment office in a province of southern Spain completed a survey about social resources, emotions, psychological adjustment, and well-being. The survey also included measures of social support, Perceived Emotional Intelligence (PEI), stress symptomatology, depressive symptomatology, life satisfaction, and happiness, along with questions related to socio-demographics variables such as age, gender, and period of unemployment. The order in which participants answered the measuring instruments was as follows: first they responded to questions related to socio-demographics variables, this was followed by the scales of social support, PEI, depressive symptomatology and stress symptomatology, life satisfaction, and, finally, they completed the scale that measures the global level of happiness. Data were collected over four consecutive months with the help of a team of research assistants. All subjects were informed that they would be asked to participate in a research study concerned with personality and emotions, and informed consent was obtained. Respondents received no financial compensation for participation in the study.

The study was carried out in accordance with the Declaration of Helsinki and the ethical guidelines of the American Psychological Association. The study protocol was approved by the Research Ethics Committee of the University of Jaén.

### Statistical analysis

Preliminary analyses were carried out to compute descriptive statistics and internal consistency, as well as to detect correlations among social support, PEI dimensions, depressive symptomatology, stress symptomatology, and subjective well-being indicators (life satisfaction and happiness). To check whether PEI dimensions are related to depressive symptomatology, stress symptomatology, and subjective well-being indicators (even after controlling for the influence of social support), we conducted a three-step hierarchical regression in which socio-demographic variables were entered first (as control variables), followed by social support, and, finally, PEI dimensions (self-emotion appraisal, other-emotion appraisal, use of emotion, and regulation of emotion).

These analyses were carried out using the SPSS package (version 20.0; IBM, Chicago, IL, USA).

## Results

### Descriptive analyses

Pearson correlations, means, standard deviations, and reliability of the different subscales used for the present sample are presented in Table 1. As expected, social support and all PEI dimensions were found to be significant and negatively related to depressive symptomatology and stress symptomatology. Similarly, social support and emotional dimensions were also found to be significant and positively associated to life satisfaction and happiness (see Table 1).

**Table 1:** Means, standard deviations, reliabilities and correlations between different measures.

	1	2	3	4	5	6	7	8	9
1. Social support	–								
2. Self-emotion appraisal	0.27**	–							
3. Other-emotion appraisal	0.26**	0.61**	–						
4. Use of emotions	0.26**	0.64**	0.51**	–					
5. Regulation of emotions	0.20**	0.61**	0.41**	0.59**	–				
6. Depression	−0.21**	−0.27**	−0.18**	−0.31**	−0.27**	–			
7. Stress	−0.10*	−0.22**	−0.13**	−0.19**	−0.30**	0.84**	–		
8. Life satisfaction	0.34**	0.29**	0.24**	0.36**	0.27**	−0.37**	−0.29**	–	
9. Happiness	0.36**	0.36**	0.25**	0.39**	0.31**	−0.31**	−0.23**	0.45**	–
M	5.72	5.36	5.36	5.28	4.95	1.08	1.42	4.35	4.96
SD	1.08	1.03	0.98	1.09	1.20	0.89	0.88	1.22	1.00
α	0.91	0.77	0.77	0.80	0.83	0.92	0.90	0.81	0.70

**Notes:**

** p ≤ 0.01.

* p ≤ 0.05.

### Hierarchical regression analyses

To examine and evaluate the separate contribution of socio-demographic variables, social support, self-emotion appraisal, other-emotion appraisal, use of emotion, and regulation of emotion for the prediction of depressive symptomatology, stress symptomatology, life satisfaction, and happiness, our study conducted a set of hierarchical regression analyses. For the first step, gender, age, and duration of unemployed were entered as control variables. Second, we entered social support: the well-known and classic dimension that is traditionally associated with psychological adjustment and the psychological well-being of the unemployed. Finally, PEI dimensions were entered into the regression. To examine whether predictors accounted for a small, medium, or large amount of the variance in psychological adjustment and well-being indicators, we used Cohen (1988) convention for small (*f*^2^ = 0.02), medium (*f*^2^ = 0.15), and large (*f*^2^ = 0.35) effects. The results of these analyses are presented in Tables 2 and 3.

**Table 2:** Results of hierarchical regression analyses of EI dimensions on depression and stress, controlling for sex, age, duration of unemployment, and social support.

Predictors	Depression				Stress			
	β	R^2^	ΔR^2^	F	β	R^2^	ΔR^2^	F
***Step 1: demographic variables***		0.06	–	10.11***		0.04	–	6.48***
Age	0.23***				0.21***			
Sex	−0.04				0.00			
Time unemployed	0.10*				0.10*			
***Step 2: traditional predictor***		0.09	0.03	8.55		0.05	0.01	5.57
Social support	−0.06				0.12			
***Step 3: dimension of EI***		0.20	0.11	12.63***		0.15	0.10	9.13***
Self-emotion appraisal	−0.04				−0.19			
Other-emotion appraisal	0.05				0.35			
Use of emotions	−0.25**				−0.05			
Regulation of emotions	−0.14*				−0.31***			

**Notes:**

*** p ≤ 0.001.

** p ≤ 0.01.

* p ≤ 0.05.

**Table 3:** Results of hierarchical regression analyses of EI dimensions on psychological well-being indicators, controlling for sex, age, duration of unemployment and social support.

Predictors	Life satisfaction				Happiness			
	β	R^2^	ΔR^2^	F	β	R^2^	ΔR^2^	F
***Step 1: demographic variables***		0.01	–	2.00		0.01	–	1.59
Age	−0.09*				−0.07			
Sex	0.04				0.07			
Time unemployment	−0.04				0.07			
***Step 2: traditional predictor***		0.11	0.10	13.75**		0.14	0.13	16.63**
Social support	0.22**				0.24**			
***Step 3: dimension of EI***		0.20	0.08	12.97**		0.25	0.11	17.17**
Self-emotion appraisal	0.03				0.13*			
Other-emotion appraisal	−0.01				−0.06			
Use of emotions	0.26**				0.22**			
Regulation of emotions	0.06				0.10			

**Notes:**

** p ≤ 0.001.

* p ≤ 0.05.

As Table 2 shows, a total of 20% of the variance was accounted for with respect to depressive symptomatology (R^2^ = 0.20; F (7, 442) = 12.63, p < 0.001). In the first step, sex (β = −0.04, p > 0.05) did not predict the depression level. However, age (β = 0.23, p < 0.001) and time of unemployment (β = 0.10, p < 0.05) reached statistical significance in predicting the depression scores. In the next step, the classic dimension associated with depressive symptomatology (social support) did not predict the depression level (β = −0.06, p > 0.05). In the final step, use of emotions and regulation of emotions had a regression coefficient that reached statistical significance: (β = −0.25, p < 0.01) and (β = −0.14, p < 0.05), respectively, explaining a small but significant amount of variance (*f*^2^ = 0.123) in the prediction of depression (ΔR^2^ = 0.11).

A total of 15% of the variance was accounted for with respect to stress symptomatology (R^2^ = 0.15; F (7, 442) = 9.13, p < 0.001). In the first step, sex (β = 0.00, p > 0.05) did not predict the stress level. However, age (β = 0.21, p < 0.001) and period of unemployment (β = 0.10, p < 0.05) reached statistical significance for predicting the stress scores. In the next step, the classic dimension associated with stress symptomatology—social support—did not predict the stress level (β = 0.12, p > 0.05). In the final step, regulation of emotions was the only factor that had a regression coefficient that reached statistical significance (β = −0.31, p < 0.001), explaining a small but significant amount of variance (*f*^2^ = 0.111) in the prediction of stress (ΔR^2^ = 0.10) (see Table 2).

As Table 3 shows, in terms of life satisfaction, a total of 20% of the variance of life satisfaction was accounted for (R^2^ = 0.20; F (7, 442) = 12.97, p < 0.001). In step one, sex (β = −0.116, p > 0.05) and period of unemployment (β = 0.042, p > 0.05) did not predict life satisfaction. Age was the only socio-demographic variable that had a regression coefficient that reached statistical significance (β = −0.09, p < 0.05). In the next steps, social support was associated with life satisfaction for the unemployed (β = 0.22, p < 0.001). In the final step, the use of emotions was the unique predictor that had a regression coefficient that reached statistical significance (β = 0.26, p < 0.001), explaining a small but significant amount of variance (*f*^2^ = 0.086) in the prediction of life satisfaction (ΔR^2^ = 0.08).

Finally, with regard to subjective happiness, a total of 25% of the variance was accounted for (R^2^ = 0.25; F (7, 442) = 17.17, p < 0.001). In step one, the socio-demographic variables did not predict happiness: age (β = −0.07, p > 0.05), sex (β = 0.07, p > 0.05), and period of unemployment (β = 0.07, p > 0.05). In the second step, social support was associated with the happiness of the unemployed (β = 0.24, p < 0.001). In the final step, self-emotion appraisal and use of emotions had a regression coefficient that reached statistical significance: (β = 0.13, p < 0.05) and (β = 0.22, p < 0.001), respectively, explaining a small but significant amount of variance (*f*^2^ = 0.123) in the prediction of happiness (ΔR^2^ = 0.11) (see Table 3).

## Discussion

Theoretical models of coping with job loss suggest that psychosocial factors may be important predictors of psychological distress and low levels of well-being (McKee-Ryan & Kinicki, 2002). Examining the personal resources that protect people or place them at greater risk for the adverse health consequences of unemployment remains an important focus for both social researchers and career counselors. The main objective of this study was to determine the unique and additional contribution of EI skills on psychological distress, depression, and well-being outcomes during unemployment, in combination with the other traditional predictor of levels of distress and well-being during unemployment (social support).

Previous research has consistently documented the detrimental effect on mental health and well-being that follows job loss (McKee-Ryan & Kinicki, 2002). In fact, unemployment has traditionally been related to lower levels of well-being and related to higher levels of stress, depression, and anxiety, among others (McKee-Ryan et al., 2005; Wanberg, Kammeyer-Mueller & Shi, 2001).

Our results have showed that social support was negatively related to depressive symptomatology and stress symptomatology and positively related to life satisfaction and happiness. Furthermore, a similar pattern was found with EI dimensions. Specifically, all EI dimensions were also negatively related to symptoms of depression and stress and positively related to life satisfaction and happiness in the unemployed. These results are in line with previous research and they extend prior work by underlining that these psychosocial resources might weaken the negative psychological consequences of unemployment (depression and stress) (Bjarnason & Sigurdardottir, 2003; Wanberg et al., 2012). Similarly, these personal resources might also promote higher levels of well-being in the unemployed (McKee-Ryan et al., 2005; Rey, Extremera & Peláez-Fernández, 2015).

Regression analyses indicated that, beyond traditional socio-demographic variables, social support was not a significant predictor of depression and stress levels. However, social support did explain a significant percentage of variance in life satisfaction and happiness. These findings are consistent with the available empirical evidence, according to which social support is one of the main sources of subjective well-being (Siedlecki et al., 2014), and also with past research on the link between social support and higher well-being in unemployed people (Rey, Extremera & Peláez-Fernández, 2015).

However, our results have shown that one of the dimensions of PEI, use of emotions, was a significant predictor after controlling for socio-demographic variables and social support, except for stress. This PEI dimension is related to a person’s ability to make use of his or her emotions by directing them toward constructive activities and personal performance. An unemployed individual who is highly capable in this ability would be able to encourage him/herself to continuously do better in coping with job loss. Furthermore, those unemployed people that have highly developed abilities to use and reason about emotions would also be able to direct their emotions in positive and productive directions. They might also be better able to control their affective reactions in response to unemployment depression and report higher life satisfaction and happiness compared to their unemployed counterparts with a low ability to use emotions. Thus, after controlling for the demographic variables and other well-known dimensions, such as social support, use of emotional skills explained a significant and additional variance in three out of four well-being outcomes. These results with the unemployed partially replicate the results obtained in previous cross-sectional studies with other samples in which use of emotions, assessed by WLEIS, was the most important predictor of all PEI dimensions in predicting depression levels (Shi & Wang, 2007), life satisfaction (Law, Wong & Song, 2004), and happiness (Khosla & Dokania, 2010).

Moreover, after controlling for socio-demographics and social support, the ability to regulate emotions was the only resource that explained significant variance in stress symptomatology. It is tentative to think that unemployed people who are highly capable of regulating their own emotions would be better able to manage stressful situation by carrying out social and cognitive strategies, for example, cognitive reappraisal and emotional acceptance, to effective manage negative emotions (Shallcross, Troy & Mauss, 2015).

Hence, it is noteworthy that the predictive power of EI abilities appears to be mostly due to the joint contribution of use of emotions and regulation of emotions. Emotional information plays a critical role in our working lives since active job searches are governed by rules of behavior that are triggered by our emotions. Being able to effectively use emotions to facilitate positive thoughts and to regulate emotions and actions may have an impact on health and well-being, making unemployed people more adaptable to daily life stressors and more able to use and repair emotions for managing conflicts and difficult situations, such as job rejections, financial adversity, and interviews, among others. Individuals with high use and regulation abilities are thought to clearly discern their moods and to access and generate emotions that assist with optimistic thoughts, motivate positive actions, and mediate negative effects and behaviors, all of which should contribute to greater levels of mental health and well-being.

Traditionally, positive psychological approaches have considered that healthy people use psychological and social resources (self-efficacy, optimism, resilience, hope, humor, etc.) to cope with stress, anxiety, and depression, as well as to increase their well-being (Seligman & Csikszentmihalyi, 2000). Our results are in line with this reasoning and extend previous findings that suggest unemployed people who score highly in emotional skills are better equipped to handle stressful situations during unemployment and to report high psychological adjustment and well-being outcomes.

In spite of its contribution to the field, this study is not without certain limitations. First, self-reports were used as a method of assessment of both EI and well-being indicators, which is not the best choice since self-reports are known to be contaminated by inherent shared method variance problems and bias of overestimation (Schutte et al., 2007). Further research with performance measures that test EI and medical health indicators for well-being and distress is needed. Our study included unemployed individuals recruited by purposive sampling methods, which is a non-random sampling technique. A limitation of adopting a purposive sampling is that a small sample size and the non-random nature of the sample may place severe constraints on the ability to generalize findings to the general population. Therefore, these findings need to be replicated with other larger and random unemployed populations in order to generalize our results. Finally, self-emotions appraisal and other-emotions appraisal (two dimensions of the PEI) were not as statistically significant as predictor as were use of emotions and regulation. One possible explanation for these weak associations could be related to the sample size; the use of a greater sample could show more robust associations. A further possibility is that this could be related to the low scores obtained by participants in depression and stress. Therefore, the use of clinical samples with high levels of symptomatology could overcome this limitation.

## Conclusions

To conclude, this research lends credence to those intervention programs that focus in part on stimulating emotional abilities to enhance coping in individuals who experience challenging and stressful situations during unemployment. Even though our research suggests that specific emotional skills might play an important role in quality of life, further research may better examine specific conditions in which EI is relevant through different stages of unemployment, and may better define what objectives and key results are more important (i.e., active job searching, employability, psychological adjustment). Including EI training in vocational guidance programs that emphasize the use of emotional skills may have the potential to improve psychological functioning during unemployment (Hodzic et al., 2015). Moreover, further mediational research should examine whether the impact of EI on employability is in part a result of improvement in mental health and well-being. Identifying such factors would also provide valuable information to both career counselors and social researchers in order to design effective assistance interventions to improve quality of life during unemployment.

## Supplemental Information

10.7717/peerj.2506/supp-1 Supplemental Information 1 Participant scores in the following variables: age, sex, marital status, time unemployment, social support, self-emotion appraisal, appraisal-emotion other, use of emotions, regulation of emotions, depression, stress, life satisfaction, and happiness.

## Additional Information and Declarations

### Data Deposition

The following information was supplied regarding data availability: The raw data has been supplied as Supplemental Dataset Files.
